# Lectures on the Principles and Practice of Physic, Delivered in King's College, London

**Published:** 1844-01-01

**Authors:** 


Lectures on the Principles and Practice of Physic, delivered at King's College, London. By *T. Watson*, M.D. London, 1843.

A System of Clinical Medicine. By *R. J. Graves*, M.D. M.R.I.A. Dublin, 1843.

We shall commence this article by a condensed analysis of Dr. Watson's Lectures.

In the Introductory Lecture, Dr. Watson describes the general order and arrangement of the course, and defines some of the terms which he shall find it necessary to use. The term *disorder*, which is generally applied to simple derangement of function, where no alteration of structure is presented, he uses synonymously with *disease*, a term generally restricted to maladies attended with appreciable change of structure. The physiological classification of diseases, viz. into diseases of the different systems, he rejects, and for a very valid reason, inasmuch as there are many forms of disorder, that affect all these systems in common, or simultaneously, and comparatively few that are confined to any one of them. The classification of diseases according to the *tissues* he discards, seeing that no useful arrangement could be formed on such a basis, the entire body being more or less pervaded by the intermixture of several of these tissues. The principle of arrangement adopted by our author is in a great measure anatomical, being founded on the anatomy of the regions—the situation of organs. Before attempting to treat of particular diseases, he gives an outline of general pathology. A knowledge of pathology comprehends, according to Dr. Watson—1, a knowledge of the material changes to which the several parts of the living body are subject—2, a knowledge of the processes or actions by which those changes are wrought—3, a knowledge of the causes which set these processes on foot; and 4, a knowledge of the consequences of the same changes, or of the symptoms they occasion. The solid parts of the animal frame may be altered in *bulk, form, consistence, in their intimate texture* and *in situation.* The fluid parts also may be changed in *quantity;* in *quality;* and in *place.* The solid parts may be altered in *bulk* without any change of texture, and in two ways; they may become larger or smaller than natural—that is, *hypertrophied* or *atrophied.* Hypertrophy is best illustrated in the muscular system. By constant exercise the muscles acquire preternatural volume, weight, and power, seemingly according to a law of the animal economy, that increase of function leads to increase of bulk—the same holds good in other tissues also; wasting of one kidney produces increased bulk in the other. The same thing happens in the case of the lungs. The law resembles one familiar to political economists, viz. that the supply of a marketable commodity is regulated by the demand for it.

Hypertrophy unattended by any change of texture—seems to depend on more active nutrition of the part. Such hypertrophy has not the nature of disease—it is, however, often connected with disease, though not itself a morbid process. Thus we have it in the hollow contractile organs, as the heart, urinary bladder, and the intestinal tube. If these instances of hypertrophy are to be considered morbid, it must be only as associated with disease. Many consider the hypertrophy as constituting a source of disease, and a cause of danger. Dr. Watson thinks that, in most cases, it is really a compensatory change, and one conservative of life. Its relation to disease depends much on its seat. In the voluntary muscles it is generally harmless, whilst in the involuntary it is connected with disease, sometimes as a cause, oftener as a consequence, sometimes as both cause and consequence. The production of hypertrophy appears connected,—1st, with certain localities, as in the case of bronchocele—2nd, with certain congenital or acquired conditions of the body; thus, in the strumous diathesis, we have hypertrophy of the upper lip, and of the extremities of the long bones—3rd, with certain habits of life, as full diet with inactivity of the body, and 4th, with the removal of certain parts of the body, as of the testicles in man and the ovaries in the female.

The Lecturer next considers *atrophy,* a condition in which parts become smaller than natural without any other alteration of tissue. This seems to depend on a diminution of the nutritive function. The alterations caused by atrophy are sometimes perfectly consistent with health. There are parts of the body destined only for a temporary purpose, and on the cessation of their especial function they dwindle and disappear. We have instances of this in the thymus gland—the supra-renal capsules—and the parts connected with the fœtal circulation. Atrophy sometimes displays itself even in fœtal life. Hare-lip, fissure of the palate, certain malformations of the heart, are instances of intra-uterine atrophy. We would consider these rather as arrests of development, than instances of atrophy. Atrophy, considered as a morbid change, is conspicuous in the muscular system. We see it in the voluntary muscles, when a limb remains a long time in a state of inaction, whether from palsy of such limb—from pain connected with disease of a joint—or from any other cause. The atrophy in most cases seems to depend on a deficient supply of arterial blood.

Some will have it that, with respect to paralysed limbs, the change in the innervation of the part tends to occasion its atrophy.—Dr. W. thinks that this can only exercise an indirect influence by reducing the supply of healthy arterial blood. *Pressure* on the large arterial trunks, or on the capillaries, so as to lessen, without completely preventing, the supply of blood, will produce atrophy. *Chronic inflammation* will sometimes produce wasting of the parts it has occupied. Various diseases, interfering with the due performance of the digestive functions—or diminishing the quantity, and impairing the quality, of the nutrient fluid, produce more or less of general atrophy.

Various parts of the body are liable to be changed in *consistence*. They may become harder than natural (*induration*), or softer (*softening—ramollissement)*. Induration of an organ may arise without any other alteration of tissue from overfulness of its blood-vessels, as in the case of the lungs or liver. Induration of the hollow organs, or of cellular parts, will arise (without any change of texture) from undue accumulation of fluids within them, as of bile in the gall-bladder, urine in its receptacle, of gases in the stomach and intestines, and lastly of serosity in the cellular tissue (œdema). It may also be the consequence of hypertrophy. Induration of an organ may also arise from the *ex*pression of its fluid, and the *compression* of its solid parts. This is well seen in the lung when it has been flattened against the vertebral column by effusion into the pleura. Blood, —or fluids separated from the blood—may fill and obliterate the natural interstices, and, by concreting, tend to solidify and harden the part—a good instance of this is *hepatisation* of the lung. Unnatural induration may also arise from the deposition or growth of irregular masses of matter within the body, differing from any of the solids or fluids entering into its healthy composition—the varieties of tubercle, cancer, and other forms of disease termed malignant, fall under this head. The opposite condition to this is *softening*, a change to which almost every tissue in the body is liable. We see it affect the brain and spinal chord, the cellular tissue, the muscles, mucous membranes ; and even the bones are liable to it, as in the disease known by the name of *mollities ossium*. With respect to the causes of *softening*, it may be said to proceed from inflammation, from disease of the arteries of the part, from insufficient sustenance—from altered qualities of the blood—the author considers, in a word, that softening may ultimately be resolved, like atrophy, into suspended or defective nutrition. The next change considered is that constituting what has been called the *transformation of tissues*, as when in the proper place of one natural tissue we sometimes find another, which last is thus *un*natural in regard to its situation, but natural in all other respects. In most cases the tissue that has been changed or displaced is in one of the two following predicaments :—Either its natural function has been for a long time suspended ; or it has been accidentally called on to fulfil a purpose for which it was not originally destined ; in the former case it gradually approximates towards cellular tissue, which at length is all that remains ; in the latter, it assumes the characters of that other tissue, whose office it has taken up. This we see is in accordance with what we know of the laws that govern the progressive development of the human body. In the embryo all the tissues commence by being cellular, and they only assume other forms and characters, each on the condition of its fulfilling some special purpose. This explains why there should be a tendency in each tissue to revert towards its primitive state, that of cellular tissue. This law pretty nearly coincides with that which regulates the hypertrophy and atrophy of parts. Again, if the nature of the original function determines, in the first instance, the nature of the tissue, we have less difficulty in conceiving how the nature of a new and accidental function imposed upon a tissue may determine the kind of transformation it shall suffer—thus, if a muscle comes to lie around and invest an unreduced joint after a dislocation, assuming the uses of those tissues which naturally inclose the joint, it becomes converted into fibrous or ligamentous tissue. There is a limit, however, to this transformation. Nerve, muscle, and gland are convertible into other tissues, but other tissues are not convertible into them. Cartilage may become bone, but never mucous membrane; mucous membrane may be converted into skin, and vice versâ; but neither into serous membrane. We have seen that special transformations take place when a tissue falls into disuse, or a new function devolves on it. All transformations, however, cannot be included within these limits. We have ossifications of arteries, of the cartilages of the ribs, and of the larynx. Such changes are said to be the effects of irritation—age, also, has some connexion with them.

The Lecturer next considers the changes of *situation* to which the solid parts of the body are liable. These changes chiefly regard the *viscera*. Thus, in the chest, a whole lung may be displaced, and compressed against the vertebral column by serous or gaseous effusion into the cavity of the pleura. The same causes may dislocate the heart, when they operate on the left side of the thorax. In the abdomen and pelvis the various forms of hernia may be adduced as instances of dislocation. Another instance of this state is that which is called *intus-susception*.

The Lecturer, after considering the morbid changes to which the solids of the body were liable, now comes to consider the morbid alterations of the fluids, especially those of the blood. The absurdity of exclusive *humoralism*, as well as of exclusive solidism, he points out very clearly. No notable alteration can occur in the solids of the body which will not soon affect its fluids in some way; whilst every important change in its fluids must lead to, or proceed from, a corresponding and proportionate modification of its solids.

The animal fluids are—the blood, the fluids that enter the blood, and the fluids that proceed from the blood. Those that enter the blood are of two kinds—1st, those by which it is renewed and enriched; and 2nd, those that enter it in order that they may be conveyed out of the body. Thus we know that the deterioration of the first products of nutrition by any cause, as unfit aliment, or impure air, will sensibly modify the blood. Some of the matters also derived from the body itself, and taken into the blood in order to be conveyed away, may directly alter and contaminate the blood; matters, for instance, absorbed from diseased parts of the body;—or matters, which, though harmless whilst only transitory or in minute quantity, become noxious when retained and accumulated in the blood, from faulty or deficient action of the organs destined to eliminate them.

The fluids that *leave* the blood are—1st, those expended in the growth or maintenance of parts; 2, those employed in aid of some definite function of the body, as the saliva, gastric juice, bile, &c.; these may be excessive, deficient, or entirely wanting; 3, those separated from the blood to be excreted, as the urine, cutaneous and bronchial excretion.

The blood itself is subject—1st, to variations in *quantity*, both in respect to the whole system, and to particular parts or organs; 2nd, in the proportions between its several proximate constituents; and 3, to great changes in its chemical composition, and consequently in its physical qualities, as seems to be the case in sea-scurvy, purpura, &c.

It has been stated that the blood may undergo alterations in *quantity*—it may be too abundant throughout the body, or only in certain parts of the body—this constitutes general or local *plethora;* sometimes called general or local *congestions*—*hyperæmia* expresses the same state. Inflammation, hæmorrhage, dropsy, all imply a previous condition of congestion. When the growth of the body has been completed, blood may be made in greater abundance than the wants of the body require. Full living, and a sedentary life, are likely to produce plethora. In such persons, the entire vascular system is preternaturally distended. When in these persons inflammation arises, it requires active treatment. Here the blood is not only more abundant in quantity, but it is also richer in fibrin, and in red particles.

The opposite to this state of general plethora is *anæmia*—or, in plain English phrase, poverty of blood. This state may be produced by repeated abstractions of blood; by impoverished food; it is also characteristic of *chlorosis*. Various forms of hæmorrhages are, also, capable of causing it. When under such circumstances blood is drawn from a vein, we observe a small clot floating in an abundance of serum. It is curious that the red particles require more time for their restoration than the other constituents of the blood; the preparations of iron, and the respiration of pure air have signal efficacy in restoring them.

We have seen that in general plethora, there is local plethora of every organ. Strictly speaking, however, local plethora should be predicated only of a part that contains more than its share of red blood. We may, however, have local plethora without general plethora; in fact, local determinations of blood are extremely common in persons in whom the mass of blood, and the proportion of its nutritive particles, have been much diminished by disease. This remarkable tendency, under such circumstances, to an unequal distribution of the blood in the capillaries, our author thus explains. A due supply of healthy blood is requisite for the due performance of the functions of the brain and nerves, and when this supply is defective, those functions become disturbed, and in their turn disturb the functions of the solids and derange the balance of the circulation. Persons endowed with great sensibility of the nervous system are known to be very liable to partial and irregular congestions of blood. This local congestion may be produced, on the surface of the body at least, by various means; by frictions, and by mechanical or chemical means. Now the congestion thus occasioned is not inflammation, but it is the first step towards it. The congestion thus produced is said to be sthenic or active. The arteries, probably, have more to do with it, in the first instance, than the veins. It is in the capillaries which are distinct from and interposed between the minute arteries and the veins that further changes are wrought, when the process advances a stage beyond mere local plethora.

For the purpose of illustrating the subject of active congestion and the various phenomena accompanying it, he describes the scene which presents itself, when the web of a frog's foot is looked at through a good microscope. (The description is taken chiefly from Kaltenbrunner.) After terminating this account of the phenomena accompanying active congestion, he observes that, as this state is the parent of inflammation, so it sometimes causes hæmorrhage, and is relieved by it. One obvious mode of remedying this congestion is the mechanical abstraction of blood from the loaded part. This, however, may sometimes do harm. Irritability of the nervous system may be aggravated by bleeding; and in proportion as the nervous functions are irregularly performed, the tendency to unequal distribution of blood in the capillary vessels increases. Besides active congestion, such as now spoken of, we have another form of this state, called *mechanical congestion;* in this form the veins alone are concerned. This sort of congestion may be purely local, as when confined to a limb, when the principal venous trunk belonging to that limb is compressed. If there be disease of the liver, such as to prevent a free passage of the blood through that organ, congestion will take place in all those parts of the capillary system from which the blood is conveyed by the veins that ultimately concur to form the vena portæ. There is another form of congestion, differing both from active and mechanical congestion; in this form the capillaries become loaded, and the course of the blood in them is sluggish without any increased velocity of the blood in the arteries, and independently of all mechanical obstacle in the veins. To this form the term *passive* has been applied. Andral calls it asthenic hyperæmia. We see instances of this in persons enfeebled by age, or disease, in whom the lower parts of the legs, the insteps and ankles, and the skin which forms the surface of old scars, are often habitually purplish, or violet-coloured. In these cases, the capillaries appear to have lost their natural elasticity; they readily dilate under the pressure of the blood, which, being thus retarded, accumulates in the part. This state may occur without any previous irritation acting on the part, or any previous active congestion. There is, however, frequent connexion between these contrasted conditions. Passive often succeeds active congestion: the vessels become dilated under the force of the active hyperæmia, and, the irritation ceasing, they do not at once recover their tone, but remain passively distended. In the production of *active* congestion the arteries are chiefly concerned; in *mechanical* congestion the veins, and in *passive* congestion the capillaries. There is every reason for believing that the internal parts are equally liable as the external to passive congestion. Thus the lungs are very subject to engorgement of their capillary vessels. This state is most effectually relieved by tonics and stimulants. One law has been ascertained with respect to active and passive congestion; viz.—that it is apt *to recur*.

*Passive* and *mechanical* congestion often exist *together*. If the capillaries of a part are much enfeebled, the mechanical effect of the gravity of the blood may suffice to bring them into a state of congestion. This explains the occurrence of a gorged condition of the posterior portion of the lungs (evinced by symptoms during life) in persons who have had no previous pulmonary disease, but have been confined for a long time to the supine position. Mechanical congestion, when it reaches a certain point, is the source of hæmorrhage, and the almost constant precursor and immediate cause of several dropsical accumulations. Blood, poor in its materials, though not deficient in its quantity, may also occasion dropsies.

Our author now comes to consider the *different modes of dying*, that is, the ways by which the vital functions of the body are extinguished. The investigation of the different modes of dying resolves itself into an investigation of the different ways in which the circulation of the blood may be brought to a stand. The brain, lungs and heart are the three grand vital organs; hence the functions they perform are called vital functions. The functions of any of the three being arrested, the functions of the other two are also speedily extinguished. But the phenomena of dying vary very much, according as the interruption begins in the one or the other of these organs.

That the heart may continue to propel the current of the blood two things are indispensable: 1st, a certain power of contraction, and 2, a certain quantity of blood to stimulate the heart. Thus there are two ways in which death may be said to begin at the heart. The respiration is entirely subservient to the circulation of the blood. The heart and lungs respond to each other. The respiratory apparatus is added in order to ventilate the blood. To this purpose two things are requisite, 1st, the ingress and egress of air; and 2, alternating movements of the chest to cause such ingress and egress. These movements are essentially involuntary, and depend on a certain state of the medulla oblongata. The respiration depends then on the nervous system—on the other hand, the action of the heart is not directly or necessarily dependent on any constant nervous influence; it goes on in acephalous fœtuses. But though the nervous influence is not necessary to the movements of the heart—further than as it is necessary to the respiration, and the introduction of nutriment, it is known that very sudden or extensive injury or shock to the nervous system may paralyse the heart. There are, then, certain states of the brain and nerves which, without directly affecting the heart, bring the motions of respiration to a pause: and there are certain states of the brain and nerves, which act directly on the heart and arrest its play. That is, there are two different ways in which death may be said to begin at the head. Sudden death caused by a want of the due supply of blood to the heart is called *death by anæmia.* The best instances of this are persons dying of sudden and profuse hæmorrhage. Another form of death, beginning at the heart, is that wherein there is a deficiency of contractile power in the heart. This is *death by asthenia.* Some kinds of poison may produce this. Sir Benjamin Brodie ascertained, upon examining the chest after death occasioned by the *upas antiar*, that the heart was not empty, but full, there being purple blood in its right, and scarlet blood in its left cavities; so that the action of the heart does not cease from defect of stimulus, but from a loss of contractile power. The state of suspended animation common to both these forms is expressed by the term *syncope*.

In death by *anæmia* and death by *asthenia* this difference may be noted —in death by anæmia the suspension of the functions of the nervous system is caused by lack of blood to the brain from the heart; whereas, in sudden death by asthenia, the nervous system is first affected, and, through it, consecutively the heart. Now with respect to the manner in which death is produced by the suspension of the respiratory function—that is, by want of due arterialization of the blood—there are two perfectly distinct modes in which this cause of death may happen.

1st. When the access of air to the lungs is suddenly denied by some direct obstacle to its entrance—(this is death by *asphyxia.*)

2. When the muscular actions required for breathing cease in consequence of *insensibility,* caused by disease or injury of the brain—(this is death by *coma.*)

The author adverts here to the improper use of the term *asphyxia,* as applied to death resulting from the nonarterialization of the venous blood. He would prefer the term *apnœa.* The internal changes corresponding with and causing the symptoms arising from the privation of air, all proceed from the prevention of the chemical alteration naturally produced in the blood, in the capillary vessels of the lungs. The venous blood now circulating becomes inadequate to sustain the functions of the several organs it now reaches. Sudden death by apnœa is not often the result of disease, it may be produced by a spasmodic closure of the rima glottidis, or by fracture or dislocation of the upper cervical vertebræ. Death by apnœa in disease is extremely common; as in œdema of the glottis and of the submucous tissue of the larynx; false membranes in the windpipe and bronchi may cause it; this kind of death may occur in pneumonia and in pulmonary apoplexy; in bronchitis, phthisis, and effusion into the pleuræ, &c. Death by *coma* is deserving of great interest. Certain diseases of the brain produce insensibility to outward impressions; the respiration becomes slow and stertorous; at length, the movements of the thorax fail—the chest ceases to expand—the blood is no longer aerated—and thenceforward the same internal changes occur as in death by apnœa.

Death by coma may sometimes be effectually obviated by a mechanical expedient. The circulation ceases because the respiration ceases, whilst the respiration ceases from the suspension of the nervous power; hence, if the nervous functions are within the verge of recovery, organic life may be sustained by *artificial* respiration, until the insensibility has passed away.

The *causes* of disease next come under consideration. Of these he gives the usual division, viz. into predisposing, exciting and proximate. He here cautions the student against confounding the terms *predisposition* and *predisposing cause.* The predisposition is a certain state of the body—the predisposing cause is what produces that state. Our author having enumerated the principal sources of disease, those, for instance, depending on the atmosphere, on diet and regimen, &c. he next enquires more nearly into the nature and mode of operation of several of them, and proceeds, in the first place, to the consideration of *heat* and *cold,* as external agencies capable of producing disease. The range of atmospheric temperature compatible with human life is very considerable. Under the burning sunshine of the Tropics, and amid the profound frost of the Polar Regions, we alike find human dwellers. In some parts of India the temperature ranges for a long time together from 80° to 100°, and even 110° of Fahrenheit: sometimes it is said to reach to 120°—these tropical climates are thickly peopled. In the Arctic countries, on the other hand, where the sun appears above the horizon for a short portion of the year only, and where the thermometer sinks to 40° or 50° below Zero, we find inhabitants indeed, but they are very few and thinly scattered. But, *for a short time*—and *under certain circumstances*—man is capable of enduring a very much higher degree of heat than the open and general atmosphere ever attains even in the hottest portions of the earth. Whether he could continue to exist, even for a little while, under a much more intense cold than ever occurs naturally on the surface of the globe, is more questionable. It has been ascertained, by repeated experiments, that the human body is capable of sustaining very high degrees of temperature, amounting even to 240° or 260°, without detriment or much inconvenience. With respect to the effects upon the human frame of a high, yet less excessive, temperature of the air, we may set down that one very constant effect of heat is that of stimulating the *organic functions* of the body; the action of the heart is much accelerated. We have evidence to the same effect in the annual changes that take place in the vegetable kingdom at a given place, the Summer renewing its foliage, the Winter repressing it. The same observation applies to those functions which animals possess in common with plants. Towards the Poles both man and the lower animals are smaller than at the Equator. On the other hand, considerable heat, when applied for some time together, has a sedative influence on the *animal functions, i. e.* upon the nervous system; causing languor and lassitude, a disinclination to exertion, both mental and bodily. There are some forms of disease distinctly traceable to heat as their cause. The effect of hot weather in promoting the cutaneous perspiration is notorious. The same influence renders the hepatic function more active. Dr. James Johnson first distinctly pointed out the sympathy or consent that obtains between the liver and the skin, under varying conditions of external warmth. Experience proves that a high atmospheric temperature, continued for some time, has a marked influence on the liver, increasing the quantity of bile that is secreted, and altering its sensible qualities: and this disturbance of function is often followed by inflammation of the gland itself. In this country we witness almost annually the effects of sultry weather in those attacks of vomiting and diarrhœa which are so common towards the latter end of Summer, and in Autumn, when the weather has been unusually hot. The Lecturer here happily illustrates the distinction between predisposing and exciting causes. It is well known that a secreting organ is never so apt to be affected by any exciting cause of inflammation, as when the process of secretion is going on actively. This undue activity of the hepatic function constitutes a predisposition to hepatic inflammation, whilst the hot atmosphere, which produces this predisposition, holds the place of a predisposing cause of the inflammation; but the exciting cause is exposure to cold; one of the most common and best ascertained exciting causes of inflammation in general.

The effects of *cold* are in many respects the direct opposites of the effects of heat. When its application is continued, it acts as a sedative on the organic functions of both animals and vegetables. This appears from the shrinking of the external parts; the superficial arteries become unable to transmit the blood in the usual quantity through the integuments. One of the earliest effects of intense cold, more especially when combined with great fatigue, is to produce drowsiness. This was strikingly exemplified in what befell Dr. Solander among the hills of Terra del Fuego. The story is given in Captain Cooke's Voyages. In many instances, before this complete torpor comes on, intense cold has a curious effect upon the nervous system, blunting the sensations, and confusing the intellect, giving to the person exposed to it the appearance of one intoxicated.

It has long been a popular, and even a professional axiom, that *sudden vicissitudes* of temperature are dangerous; that *a previous hot state of the body* augments the hurtful effect of cold, whether applied externally or internally. This broad proposition however requires limitation. If the power of evolving heat, which is known to be inherent in the system, be entire and active and persistent—if it have not been weakened by debilitating circumstances—no danger need attend even violent alternations of external temperature. Unusual heat of the body at the time when the cold is applied, so far from implying danger, is really the condition of safety, provided the heat is steady and permanent. We know that the cold affusion has been employed in the hot stage of fever, and especially in scarlet fever, not only with impunity, but with great benefit to the patient. The same holds true when the body has been heated by exercise—provided always, that the cause remains steadily in action, that there is no local disease, and that the body is not fatigued, and fast losing its heat. The more correct statement respecting the application of cold is, that it is dangerous—not when the body is *hot*—but when the body is *cooling after having been heated*; and this principle applies whether the cold be applied externally or internally, to the surface of the body or to the mucous membrane of the stomach. Very many instances are recorded of death taking place after a copious draught of cold water.

Among the circumstances which favour the morbific effects of cold, and relate to the condition of the body itself, is to be included whatever has the effect of weakening the system, and so diminishing its capability of evolving heat, as fasting, evacuations, fatigue, &c. These lessen the vigour of the circulation, and depress the power of generating heat. The power of evolving heat is very feeble in very old persons, and in the newly-born. The bad effects of cold depend partly on the intensity of the sensation it produces—but still more on the duration of that sensation. Cold is more likely, *cæteris paribus,* to prove injurious when it is applied by a wind, or current of air, and also when it is accompanied by moisture. Certain circumstances have been noticed as counteractive of the effects of exposure to cold; as passions engaging a close attention to one object; diminished sensibility, as in maniacs, and the power of habit. *Sleep* is enumerated among the conditions of the body which diminish its power of resisting cold. Now while we sleep, sensation is in a great measure suspended, and this seems to furnish a contradiction to the principle, that the effect of cold on the health depends upon the strength and duration of the sensation excited by it. This difficulty has been disposed of by stating that the sleeper who thus suffers really does feel, and is conscious of, the sensation of cold.

Closely connected with the effects of temperature on the health is the influence of the different *seasons*. It is well known that the general health of the community fluctuates with the changing seasons. Pectoral complaints are most apt to commence or grow worse in Winter and Spring; whilst bowel-complaints are most distressing in Summer and Autumn. Among the other states of the atmosphere which may be considered as a cause of disease, *impurity* of the air may be enumerated. It is however as a predisposing influence that impurity operates. It never *generates*, according to the author's belief, continued fever; yet it will most certainly aggravate the symptoms, favour the propagation, and increase the mortality of that and other diseases, in a great degree—impure air, he considers, is a very powerful agent in calling scrofula into action, and in aggravating the strumous diathesis. Among the predisposing causes of disease is classed *hereditary tendency*. To mention some of the diseases which occur in this way, scrofula, gout, mania, and, according to some, spasmodic asthma. There can be little doubt that the circumstances of the parents do influence the physical characters of the children: it is matter of daily observation; and one of the best possible illustrations of the fact is to be found in what are called family-likenesses.

The subject of *symptoms* is next discussed. Symptoms he defines to be "every thing or circumstance happening in the body of a sick person, and capable of being perceived by himself or others, which can be made to assist our judgment concerning the seat or nature of his disease, its probable course and termination, or its proper treatment." This definition expresses the three purposes for which the study of the symptoms is cultivated, viz.

1st. To ascertain the nature and seat of the disease, *i. e.* to form the *diagnosis*.

2d. To enable us to foretell the probable issue of the disease, or to frame the *prognosis*.

3d. To direct the treatment.

Notwithstanding the great importance of a correct diagnosis, the author justly observes that almost all we know concerning the proper treatment of the sick is *originally* derived from observation, *not* of the *nature of diseases*, but of the *effects of remedies*. There have been various divisions of symptoms. Those symptoms or combinations of symptoms which distinguish the place and nature of a disease, are called *signs of disease;* those which teach us what to do, are called *indications of treatment*. A difference has been pointed out between *symptoms* and *signs*. Signs are deduced from symptoms by arranging and comparing them, and noticing the circumstances under which they occur. Symptoms are obvious to all persons alike—to the nurse as well as to the physician: signs, for the most part, are such to medical eyes only. There are other general divisions of symptoms. Some are called *direct*, some *indirect* symptoms. Direct symptoms lead to the very part affected; indirect symptoms are such as "declare themselves through the medium of some other parts, or through the medium of the constitution at large." With respect to the symptoms which consist of *morbid* changes, they may all be classed under three heads:—1. Uneasy, unnatural, or impaired *sensations:* 2. Disordered, or impeded *functions:* and 3. Alterations of structure or of appearance; changes of *sensible qualities.* When these last come within the direct cognizance of our senses, they are called *physical signs.* Of all the uneasy sensations *pain* is the most important. On finding that pain or uneasiness is complained of in any part or organ, our next business should be to enquire whether the *functions* of that part or organ are disturbed or suspended. The functions of the brain and nerves—of the heart and blood-vessels—of the respiratory apparatus—and of the digestive organs—are all of vital consequence.

The symptoms drawn from the functions belonging to the *circulation* are of the utmost importance. Every one knows how much value is attached to the quality of the pulse. The qualities most attended to in the pulse are its frequency, regularity, fulness, and force.

Our author next considers the important subject of *Inflammation;* its morbid and salutary effects, together with its local and constitutional phenomena, as it occurs in external parts. After examining the symptoms of inflammation, as Pain, Heat, Redness, and Swelling, and the different conditions on which they depend, he then considers the state of the capillaries, and of the blood in a part inflamed. Much has been learned on these points by patient and minute observation with the microscope, and by reasoning on the facts thus brought to light. In order to comprehend the minute phenomena of inflammation, it is necessary to have a clear conception of the constituent elements of the blood, and of the principal changes it is liable to undergo. The blood is known to consist of red particles, or globules, and of a transparent colourless fluid, called lymph or liquor sanguinis. The liquor sanguinis separates by coagulation into two parts, serum and fibrin, the latter having previously existed in solution in the liquor sanguinis. From the experiments and observations of Kaltenbrunner it appears that various stimulant substances, mechanical or chemical, when applied to the web of a frog's foot, will produce irregular disturbances in the circulation, which irregular disturbances are not to be confounded with true congestion; nor are they to be confounded with the phenomena of *inflammation,* which are always preceded by those of true congestion. Kaltenbrunner found, also, that (just as in congestion,) a certain interval of time generally occurred between the application of the exciting cause and the apparent development of the inflammation. This, our author remarks, accords with what we observe to be the case in respect to local injuries, and to those local internal inflammations so apt to be produced by exposure to cold. There is a pause before the mischief lights up; there is, in fact, a period, during which the inflammation seems to be hatching, and it is hence called the period of *incubation.*

On looking then at the web, to which some violence had been done, Kaltenbrunner observed, that, when the period of incubation was over, an afflux of blood took place to the part about to be inflamed; the velocity of the blood in the vessels was greatly increased; the vessels themselves were distended and tense, and therefore disposed to tighten on the blood they contained—the functions of the part, that is to say, the secretion and absorption of lymph, were interrupted ; the blood underwent an evident change—or it failed to undergo the proper changes ; its globules stuck together, and the parenchyma of the web became tumefied—this is what constitutes the state of the blood-vessels under *active congestion,* which congestion is just one step short of inflammation. The congestion, now described, encreases, until at length the capillary tubes, instead of tightening upon their contents, become dilated ; the circulation, at first so rapid, begins to be *delayed* in some of the capillaries ; the direction of its motion becomes uncertain ; it oscillates, as it were, irregularly in those vessels, and at last stops altogether, the globules cohering in irregular masses, and thus *points of stagnation* are formed. Around these points, beyond their circumference, the circulation remains still very rapid, and the congestion persists. This is *inflammation*—of which the characteristic or pathognomonic feature is the formation of these *points of stagnation.* One early consequence of the stagnation of the blood is, that a portion of it transudes through the sides of the vessels containing it : the serum ; or the liquor sanguinis ; or even sometimes the blood itself, red particles and all. The effused serum remains, or is absorbed, as serum. The fibrin, when it has so transuded, concretes, and thus the interstices of tissues are filled up, and layers of coagulated lymph are formed on the surface of inflamed parts, constituting false membranes. Microscopic investigation has recently discovered a number of *colourless corpuscles* floating in the liquor sanguinis. These corpuscles, passing into the interstices of the inflamed tissues, or stagnating in its capillaries, suffer remarkable changes, assume a yellow colour, and are thus transformed into globules of *pus.* So that pus is nothing else but altered blood. During the inflammatory state the corpuscles sometimes appear to multiply with great rapidity. Whether the colourless corpuscles be independent of the red globules of the blood ; or whether, as some suppose, they are originally derived from the red particles, are questions as yet undecided. In fact most of the events or consequences of inflammation are traceable to the stagnation of the blood in the capillaries, and to the changes which the stagnant blood subsequently undergoes.

Our author next considers the various causes which have been assigned by pathologists for the occurrence of the *buffy coat* of the blood. That the formation of this coat depends upon some *vital* change in the blood, appears probable from this—that it will sometimes vary greatly in different portions of blood abstracted at the same bleeding. The explanations that have been offered, however, of the phenomenon, are purely hypothetical. The author dwells the more on this peculiar appearance of the blood from its great importance in determining the nature of various complaints, and in directing our treatment of them. Speaking generally, when a given organ is inflamed, the buffy coat is more marked in proportion to the intensity of the inflammation ; when the organ is not known, it is more likely to be of a fibrous or of a serous texture, in proportion as the blood is more decidedly buffed. The appearance of the buffy coat is especially valuable as an indication of treatment in cases concerning which we are in doubt whether they are inflammatory or not. On the other hand, if other symptoms indicate the presence of inflammation, the absence of the buffy coat should not shake us in our opinion. This appearance is not unfrequently absent in inflammation of the mucous membranes, especially in that of the mucous lining of the bronchi. Buffy blood is not confined to cases of inflammation—it is often found in cases of general plethora, and also in cases of pregnancy.

Buffy blood is no measure of the *danger* of the disease. Nor is the appearance of buff on the blood, taken by itself, a sufficient warrant for abstracting more blood: for the blood will sometimes, in common inflammation, continue to be buffy, long after it has ceased to be useful, or safe to bleed the patient.

Our author coincides with those who object to the word "*terminations*" of inflammation as inappropriate; alleging, that the inflammation does not necessarily cease or terminate whenever these so-called "terminations" happen. Some of them are in fact co-existent states, or successive stages in the progress of the same inflammatory disease. Some have proposed to speak rather of the *local effects* of inflammation; but even this phrase he considers not altogether unobjectionable, for sometimes (though rarely) there are no local effects produced beyond the four symptoms which characterise the inflammation itself—he prefers the expression *events of inflammation*. The events of inflammation are, Resolution—Effusion of serum —Effusion of coagulable lymph, and Suppuration.

According to the most recent observers, pus is altered blood. It is an opaque, smooth, yellowish fluid, of the consistence of cream, and having no smell—such are the characters of healthy pus. It consists of yellowish globules, diffused through a thinner fluid, which resembles in some respects the serum of the blood. "If six or eight ounces of good pus be suffered to stand in a phial, it will separate into two portions; a yellowish matter will sink to the bottom, and there will be a slightly yellow, clear, supernatant fluid, like oil in appearance, but not greasy to the touch." The sediment consists of the globules; which Gendrin supposed to be the red globules of the blood altered; deprived of their coloured envelopes, and swollen or enlarged. The opinion, however, now prevalent among pathologists is, that these pus-globules are transmuted "colourless corpuscles"—moreover they are hollow cells. In the generality of cases the formation of pus is preceded by the effusion of coagulable lymph, with or without the effusion of serous fluid; the pus in these cases appears to be poured fourth or secreted by the coagulable lymph after it has become organised. Its formation seems to characterize a more advanced stage of inflammation —to denote that the inflammation has been pressed a little beyond the adhesive stage. This is the opinion of both Hunter and Gendrin. The air possesses considerable influence in promoting the suppurative process in preference to the adhesive. In simple pleurisy from exposure to cold, we seldom have any fluids effused, except coagulable lymph and serous fluid. But if the inflammation has been caused by a punctured wound from without, or by laceration of the *pulmonary* pleura by the sharp end of a fractured rid, or by a perforation of the pulmonary pleura by the extension of a vomica in the lung—in all which cases air finds its way into the cavity of the pleura—the true *empyema* results—pus is formed. So also in pneumonia; at first the inflamed lung is rendered solid by the effusion of coagulable lymph into the air-cells; but, if the inflammation persists, the next thing that happens is what is called by Laennec *gray* hepatization—

a puriform infiltration takes the place of the lymph. In general it may be said of surfaces that are open to the air, of tegumentary membranes, that either pus is formed upon them, under inflammation, without any previous effusion of plastic lymph, or the lymph is slight in amount and transient in duration, and presently superseded by a puriform discharge.

A *fifth event* of *inflammation* is *ulceration*. Kaltenbrunner observed the progress of absorption in the inflamed tissues which he examined by the help of the microscope. Independently, however, of these microscopical observations, it is quite evident that absorption goes on, often very actively, during the continuance of inflammation. The effused fluids, or products of inflammation, the serum, the lymph and the pus, are partly taken up again; and not only are these products of inflammation liable to be removed, but the original textures of the body are carried off by absorption. We have a proof of this in the progress that an abscess makes to the nearest surface at which the pus it contains may be discharged.

Many circumstances influence the occurrence and progress of ulceration; and great differences are observable in the different tissues in respect to the facility with which they severally ulcerate. This process is most common in the tegumentary membranes. It occurs frequently in the inner coats of arteries, in cartilages and in bones; whilst it is rare in fibrous tissues of all kinds, in serous membranes, and in the outer coats of arteries. Ulceration may lead to perforation of the alimentary canal—of the air-tubes—of the gall and urinary bladders—of the blood-vessels; and to the fatal escape of the natural contents of these organs. There are three things going on at the same time in an ulcerated surface; 1, effusion of plastic lymph by which granulations are formed—2, suppuration, and 3, absorption.

The next event of inflammation noticed by our author is *mortification*. This, with respect to internal parts, occurs more frequently in some than in others. It is frequent in cellular tissue; and in the mucous and submucous tissues of the alimentary canal; in the throat, for example, in cynanche maligna; and in the glandular parts of the intestines in fever. It seldom affects the other mucous systems—those which belong to the air-passages and the urinary organs. It occurs sometimes, but not very often, in the substance of the lungs—seldom in serous and fibrous tissues. The occurrence of mortification depends on various causes and conditions—sometimes on the mere intensity of the inflammation—whatever tends to enfeeble the circulation in the part affected, or in the system at large—tends to promote it. Mortification may be produced by other causes, as well as by inflammation—the mere cutting off the supply of arterial blood, independently of any inflammation, will cause it. Ossification of the arterial trunks, and consequent stagnation of the blood in them, is the commonest cause of the dry gangrene of old persons—the *gangrena senilis*. The author now comes to consider the constitutional symptoms of inflammation. Inflammation sufficiently extensive to disturb the general system at all, is attended with pyrexia; and the presence of pyrexia, when the part affected is unseen, marks the *nature* of the disease. The most prominent of the symptoms denoting *inflammatory fever* are debility and chilliness, followed by, or alternating with, increased heat of skin; and increased frequency and force, and often *hardness* of the pulse, with considerable derangement of most of the natural functions of the body. There is generally head-ache and confusion of thought, languor, thirst, loss of appetite, a furred or white tongue. This febrile condition generally *succeeds* the manifestation of the local symptoms of the inflammation, and should then be considered as the *effect* of the inflammation.

When inflammation, external or internal, has gone on to the formation of pus, that event is marked by the supervention of peculiar symptoms; and the character of the fever is changed. The *commencement* of suppuration is often marked by rigors; and its continuance by hectic fever. The leading symptoms of *hectic* fever are an abiding frequency of pulse; alternations of chilliness with heat and flushing, followed by perspiration; a gradual wasting of the body, and progressive debility.

With respect to hectic, considered as an indirect symptom that suppuration has succeeded to inflammation, and is still going on, we may notice the strong contrast it presents, in many particulars, to the *inflammatory* fever that attends the earlier stages of inflammation. The pulse loses much or all its *hardness* and strength; but it remains permanently more frequent than the pulse of health; the appetite returns in a great measure; the thirst abates; tongue becomes clean and moist, and towards the end is sometimes unnaturally red, or speckled with aphthæ—face usually pale; but during the exacerbations it is partially flushed, and very often a characteristic red spot appears on either cheek. Emaciation, with various minor changes marking the want of proper nourishment.

The notion that hectic fever resulted from the re-absorption of pus into the blood, was at one time very generally entertained; but there are many facts opposed to this opinion. Considerable collections of matter frequently disappear, without occasioning the slightest approach to hectic. Several facts go to prove that hectic is not simply a consequence of the absorption of pus into the blood. It was the opinion both of Hunter and of Abernethy, that sympathetic hectic fever is a *teased* action of the system, endeavouring to throw off what annoys it; the cause of irritation being removed, it ceases forthwith. That there is such a thing as *idiopathic* hectic, hectic unconnected with suppuration any where, cannot be doubted. A form of fever not to be distinguished from hectic, is often seen to occur in mothers who have suckled their infants too long; it is seen sometimes in newly-married husbands; and likewise in persons labouring under diabetes. What is common to all these cases is, that there is an habitual drain upon the system beyond what the nutriment taken into it can supply and counterbalance. It is certain too that hectic fever sometimes happens in phthisis, not only before there has been any expectoration of puriform matter, but prior even to the softening and suppuration of a single tubercle. The fever attending mortification as an event of inflammation, generally puts on the typhoid form; it is characterised by sinking of the pulse, shrunken features, coldness and clamminess of the skin, a dry and black tongue, low muttering delirium or stupor, tremors of the voluntary muscles, with spasmodic startings of their tendons, and insensibility to the passage of the fæces and urine. One circumstance sometimes indicating the supervention of internal mortification is sudden cessation of pain.

The author having now concluded his remarks on the local and constitutional events of inflammation, next proceeds to consider the modifications of inflammation according as it affects the *different tissues* of the body.

When inflammation attacks the *cellular tissue*, there is a strong tendency to form circumscribed abscesses; the extension of the suppuration is prevented by a wall of lymph built up around it. The adhesive inflammation sets bounds to the suppurative. When no such boundary is set up, but the inflammation spreads, we have what is called *diffused inflammation of the cellular membrane.*

The substance of the *glands and solid viscera* suffers changes similar to those observed in the cellular tissue. The cellular tissue is liable to be rendered thick by *chronic* inflammation, as well in the parenchyma of organs, as where it is spread out beneath the skin, or beneath serous and mucous membranes. Chronic induration and thickening of the cellular tissue composing Glisson's capsule, is no unfrequent result of slow inflammation, producing that particular change of the liver called *cirrhose*, the essence of which is atrophy of its lobules from compression of its nutrient arteries.

The inflammation of *serous membranes* is characterized by sharp pain; by hardness of pulse and buffy blood; by its tendency to spread; by the effusion of serum and of coagulable lymph; and sometimes (when air is admitted), by the effusion of pus. *Adhesive inflammation* occurs in this tissue. *Ulceration* commencing in a serous membrane is very uncommon, as is also mortification.

The *synovial membranes* have a strong analogy with the serous. They are, however, less liable to inflammation than the serous—they seldom throw out coagulable lymph, and therefore adhesion of their opposite surfaces is uncommon. Inflammation of the synovial membrane speedily leads to a *serous effusion* into the joint; which often, especially in rheumatism, is as speedily taken up again.

When the *tegumentary membranes* (the skin) are inflamed superficially the inflammation is characterised by a diffused red blush only, which may be banished for a moment by pressure with the finger,—it terminates speedily in resolution, the only consequence of the inflammation being the removal of the cuticle in small branny fragments—*desquamation.* The superficial inflammation is called *erythema.*

Inflammation of the *internal* tegumentary membranes,—of the three internal surfaces which communicate with the air, and are clothed with *mucous membrane*, is very interesting. The first peculiarity which we shall notice in them is their indisposition to adhesive inflammation—the wisdom and beauty of this provision are obvious. Inflamed mucous membrane may throw out serous fluid—viscid mucus—pus, or blood. Inflammation of these membranes, however, is sometimes attended with the exudation of something very like coagulable lymph. The tracheal, bronchial, and pulmonary mucous membrane, the œsophageal, the intestinal, and that which lines the uterus, are all more or less subject to the formation of adventitious membranes under inflammation. These membranous exudations never become organized. They appear to consist of inspissated and altered mucus, and consist, in a great measure, of albumen.

Dr. Watson having now considered the phenomena of inflammation, local and general; its symptoms, and its events; and the intimation of those events afforded by the state of the system at large; having surveyed also the principal tissues of the body, and observed the modifications and peculiarities to which the process of inflammation is liable, according to the tissue affected, next proceeds to consider certain *varieties* of inflammation; viz. the acute and chronic; latent and specific, scrofulous, &c. In his account of the origin of tubercles; of the forms they assume; of the phenomena attending their enlargement, and subsequent softening, he is a close follower of Dr. Carswell.

The question has been much discussed whether the deposition of tubercular matter be not an *event* of inflammation—in Dr. Watson's opinion there is not a shadow of evidence to show that the deposit of tubercular matter is always and necessarily preceded by inflammation. Still he admits that an undoubted *connexion* obtains between the occurrence of inflammation and the occurrence of tubercles. Tubercles will cause inflammation, and inflammation will determine the development of tubercles. The enlarging tubercles excite inflammation in the surrounding textures by the pressure they exert on them, and probably in other ways; the inflammation lit up is usually of the scrofulous kind; it is slow, partial, and easily *quieted* by treatment, though scarcely to be *cured*. On the other hand, there are good reasons for thinking that, in a person having the scrofulous diathesis, the occurrence of inflammation within the chest may rouse that previously dormant tendency into action, and become the exciting cause of the separation of tubercular matter from the blood. The connexion between tubercles and inflammation is shewn also by their occurrence in the substance of false membranes.

We now pass on to the *Treatment of Inflammation*, and to the remedies recommended by the author; after pointing out the advantages of blood-letting, and purging, he next proceeds to consider the use of *mercury;* on the employment of which, he makes some excellent practical remarks.

According to Dr. Watson, the great *remedial* property of mercury is that of stopping, controlling, or altogether preventing the effusion of coagulable lymph. Thus it is chiefly useful in common adhesive inflammation, as an auxiliary, however, to blood-letting, not as a substitute for it. On the other hand, it is likely to do harm in those cases, "where the morbid action approximates to its own action,"—in cases of erysipelatous inflammation having a disposition to gangrene; in scrofulous diseases; in inflammatory diseases attended with general debility, and an irritable condition of the nervous system. When we have to contend with acute inflammation and desire to arrest the deposition of coagulable lymph, our object is, after premising the necessary bleeding, to bring the system as speedily as possible under the specific influence of mercury. Now this is best effected by giving some form of mercury in equal and repeated doses, by the mouth. For urgent cases, calomel is the best form of mercury; two or three grains, given every four or six hours, will generally suffice to touch the gums in 36 or 48 hours. If it should act as a purgative, add a little opium. Previous bleeding renders the body more readily susceptible of the influence of mercury. Considerable caution is required in the employment of mercury in consequence of certain idiosyncracies which are found to exist in different individuals. Some persons are found to be extremely susceptible to the influence of mercury; in them, very small quantities of mercury are found to act as a violent poison, a single dose producing in them very severe salivation, and very distressing effects on their mouth. For the relief of this latter annoyance, Dr. Watson recommends the following treatment. If there be much external swelling, treat the case as one of *local inflammation :* Apply eight or ten leeches beneath the edges of the jaw-bones, and wrap a soft poultice round the neck, into which the orifices made by the leeches may bleed—in nine cases out of ten this will afford the utmost relief. Pure tannin, moistened and smeared upon the spongy gums, is remarkably efficacious in rendering them firmer and more comfortable. When the flow of saliva and the soreness of the gums form the chief part of the grievance, the Doctor has found nothing more generally useful than a gargle made of brandy and water. An observation made by the author in this place is well worth remembering, as being of practical value. He mentions an instance of a very fashionable and successful physician, now dead, who used sometimes to say, when he met others of his brethren in consultation, " It is all very well to speculate about the exact situation, and the precise nature of the disorder, but the question with me is, what is good for this, that, or t'other thing." A wise physician will seek to combine with an accurate knowledge of disease those practical expedients and minor appliances which are picked up by casual experience, and which could never have been reasoned out.

Mercury is of great use also in *chronic* inflammation. The treatment, by mercury, however, should *keep pace* with the disease. When textures have been slowly altered by a gradual deposition of coagulable lymph, little is to be gained by rapidly salivating the patient. The lymph, if it can be suppressed at all, must be *gradually* taken up again: and mercury, given with the view of promoting its absorption, must be slowly and gradually introduced into the system. The greatest caution is necessary in the exhibition of mercury in scrofulous inflammations.

Another remedy employed for the relief of acute inflammation is Antimony. This substance, according to the author's experience, is admirably suited to cases of active inflammation, in which mercury would either be not so useful, or could not be brought to bear. It is in inflammation of the mucous membrane of the air-passages that antimony is so signally beneficial. It does not seem to be nearly so valuable a remedy as mercury, when serous membranes are inflamed.

There is another drug much employed in the treatment of inflammation, and that is opium. The employment of a full dose of opium after a full and effective bleeding has been found very beneficial; it is said to possess the power of preventing the rekindling of the inflammation which is apt to result from irritation of the nervous system—a kind of irritation, which the copious abstraction of blood is calculated to produce. The opium soothes this irritability; and when given for this purpose, it must be given in full doses. This remedy, however, requires considerable discrimination in its employment. Where there is active inflammation within the cranium, its propriety is very questionable. It is not devoid of danger in pectoral complaints. In inflammatory diseases which tend to produce death by *coma* or *apnœa*, great caution is necessary in its use. If there be any unnatural duskiness of the face, if ever so slight a tinge of purple mingles itself with the red colour of the lips, opium should not be employed. These appearances show that the blood is but imperfectly arteterialised, and imperfect arterialisation of the blood conduces to the state of coma. It is in cases where the tendency is toward death by *asthenia,* that the use of opium, as a remedy for inflammation, is most serviceable. It has an excellent effect often, after free bleeding, in cases of peritonitis and of enteritis; it seems to quiet the nerves—sustains the faultering action of the heart—and keeps the inflamed parts at rest.

Our author next proceeds to the consideration of *Hæmorrhage,* and this form of disease he considers only as it falls under the cognizance of the physician. Hæmorrhage, when it takes place from the unbroken surfaces of organs, without any appreciable lesion of arteries, veins, or capillaries, is said to occur by *exhalation.* In this way most of those varieties of hæmorrhage which come before the physician, take place. There are several persons who are subject, during the greater part of their lives, to discharges of blood, which happen again and again, commonly at regular intervals, without any perceptible detriment to the general health, independently of any obvious exciting cause: these are called *habitual hæmorrhages.* Again, there are certain forms of hæmorrhage, not habitual, which may be denominated *idiopathic,* in as much as they are apt to arise without any perceptible connexion with antecedent local disease. Two forms have been noticed of idiopathic hæmorrhage; viz. *active* and *passive* hæmorrhage. Active hæmorrhage is preceded by active congestion, and therefore is akin to inflammation. Passive hæmorrhage often occurs without any apparent previous congestion of any kind—this has been ascribed to morbid relaxation in the vessels or apertures through which the healthy exhalations are transmitted. A more probable hypothesis is, that such hæmorrhages are to be ascribed to some change in the consistence or composition of the blood. By far the greater number of hæmorrhages by exhalation are *symptomatic;* that is, they result from some previous disease, either in the organ from which the blood proceeds, or in some other organ connected therewith by community or dependence of function. These secondary, or symptomatic hæmorrhages, are preceded by congestion, but, for the most part, the congestion is not of the active, but of the mechanical kind, and has more to do with the veins of the part than with the arteries. Thus we have hæmorrhage from the bronchial membrane in consequence of crude tubercular matter in the lungs, filling up a portion of the pulmonary tissue, and obstructing the circulation of the blood through it. Again, we have hæmorrhage into and from the lungs, as a consequence of such disease of the heart as mechanically impedes the return of the blood from the lungs to that organ; a narrowing of the mitral orifice, for example. Hæmorrhage by exhalation occurs most frequently from *mucous* surfaces; as from the mucous membrane lining the nasal cavities; from the pulmonary mucous membranes; from the stomach and bowels; from the urinary organs, and from the uterus. It is not, however, from mucous surfaces alone that hæmorrhages by exhalation may take place; they may occur, but more rarely, from serous membranes.

Our author now directs his attention to the important subject of *Dropsy*, by which he means a collection of serous liquid in one or more of the shut cavities of the body, or in the cellular tissues, or in both; independent of inflammation. Dropsy being said to be rather a symptom of disease, than a disease in itself, it has appeared to some more scientific to treat of the original malady, than of its effect. Dr. Watson disapproves of this view of the matter, for these reasons;—1st. Because it is often uncertain, whilst the patient is still alive, what or where the primary disease may be; and, even after death, it frequently happens that no organic disease is discovered sufficient to account for the effusion; in such cases the dropsy is the disease, and the only object of treatment: secondly, dropsy is, to a medical eye, in all cases, something more than an effect or symptom; the imprisoned liquid is often the cause of other symptoms—the removal of the dropsy, it is well known, will restore the patient to comparative comfort.

In dropsy depending on organic disease, two sets of symptoms are to be distinguished; *scil.* those depending on the primary disease, and those depending on the collected fluid. When the cells or cavities which become the seat of dropsical effusion, become distended with the serous fluid, which they habitually secrete, one of these things must have happened; either the quantity of the fluid exhaled must have been increased, the absorption remaining the same; or the absorption must have been diminished, the exhalation continuing the same; or else the exhalation has been increased, whilst the absorption was either lessened, or not proportionally increased. It is only lately that the direct agency of the blood-vessels in the production, as well as in the removal of dropsies, has been recognised. Pathologists, even at the present day, speak of want of energy of the absorbents as a cause of dropsy. But certainly there is no deficiency of absorbing power in persons labouring under dropsy; they are thin and very much emaciated; and, further, they are as susceptible of the action of mercury as other people. According to Dr. Watson's views, as well as those of other pathologists, the function of absorption is shared among the lacteals, lymphatics and *veins*—the lacteals absorbing the chyle from the surface of the alimentary canal, and conveying into the blood the materials of its renovation, while the lymphatics take up and carry into the blood those old and effete portions of the solid constituents of the body, which require to be removed to make way for a fresh deposit; the office of the veins being to imbibe the serous fluid exhaled from the surfaces, membranes, and into the meshes of the cellular tissue. If this view be correct, all difficulty vanishes. Of the two sets of absorbing vessels, the lymphatics and veins, one set may continue to perform its functions, whilst the other fails. We know, from Magendie's experiments, that the *veins* perform a large share in the whole process of absorption. It has also been proved that fluids may and do pass into or out of the veins, in the living body, by mere physical imbibition and transudation, through the coats of these vessels; that, when the veins are distended to a certain degree with watery fluid, the entrance of more of the same fluid, through their sides, is prevented; that, when the distention is still greater, the aqueous part of the blood may even pass in the other direction out of the vessel; and that, on the other hand, when the veins are comparatively empty, the surrounding serous fluid passes readily into them, or, in common language, is absorbed. The venous absorption is explicable, therefore, on the principles of *endosmose* and *exosmose*.

According to these views, there can be no difficulty in showing that the chronic forms of dropsy are attributable, partly, and chiefly, and in many instances entirely, to undue plenitude of the veins, which plenitude is produced, almost always, by some impediment to the free return of the blood towards the heart.

Now, the nearer to the heart this impediment to the free return of the blood is placed, the more extensive is the venous repletion, and consequently the dropsical accumulation. And, if we could plant an obstacle at the very termination of the venous stream, we should dam up the blood in the whole system of veins, and produce general dropsy. Now such an obstacle is frequently placed there by disease. The returning blood is checked at its entrance into the heart; at the confluence of all the veins of the body, where they unite to empty themselves into the right chambers of that organ; and then anasarca of the universal cellular tissue comes on, and water collects in all or most of the great serous cavities. Sometimes we find dilatation of the right chambers of the heart, and the tricuspid valve unequal to its office of closing the aperture between them —or the retarding cause may be in the left side of the heart, keeping the pulmonary blood-vessels unduly full, and thereby hindering, indirectly, the escape of the blood from the right ventricle. So that the dropsy may ultimately depend upon some bar to the circulation, placed even at the mouth of the aorta.

Anasarca frequently occurs without any obliteration of veins, and independent of any cardiac disease. This is seen every day in weak chlorotic girls, with bloodless cheeks, and pale lips. Still, even in such cases, there is really a retardation of the venous circulation, not by any mechanical obstacle opposed to the venous circulation, but from the debility of the heart, whose office is to propel the blood onwards with a certain degree of force. In girls of this description the voluntary muscles are weak and flabby, and it is reasonable to presume, that the involuntary muscle, the heart, partakes of the general debility of the muscular system, and becomes incapable of sending the blood forwards with sufficient energy. Andral mentions a certain *cachectic* disposition of the body to be a cause of dropsy; persons may be bled into a dropsy, and starved or weakened into a dropsy. Dropsies, whose cause is traceable to the heart, are called *cardiac* dropsies. Another class of dropsies, connected in a remarkable manner with certain diseased conditions of the kidneys, are styled *renal* dropsies.

Another source of dropsical swelling is excessive *exhalation*. Dropsy, so caused, comes on suddenly, and is termed *acute* or *active*. To understand this class of dropsies, we refer to physiology. Besides the constant exhalation from the inner faces of the shut serous cavities, a large amount of watery fluid is continually thrown out of the system by the skin, the lungs, the bowels, and the kidneys. Now, when the excretion of aqueous fluid from one such surface becomes checked, the exhalation from some other surface becomes augmented. This compensating relation is more conspicuous between some parts than others. It is very evident between the skin and the kidneys. In warm weather, when the perspiration is abundant, the urine is proportionally scanty and concentrated. But, supposing the exhalation from one surface or organ to be much diminished without a corresponding increase of function in the related organ, or in any excreting organ communicating with the exterior, then dropsy is very apt to arise. The aqueous fluid thus detained in the blood-vessels, seeks, and at length finds, some unnatural and inward vent, and is poured forth into the cellular tissue, or into the cavities bounded by the serous membranes. Dropsy of one part or organ supervenes suddenly on the rapid disappearance of a watery collection from another part. We not uncommonly see the swollen legs and thighs of an anasarcous patient quickly unload themselves, and resume their natural size and symmetry; but, as his legs are emptying, he becomes drowsy, comatose, apoplectic, and, after death, the ventricles of the brain are found distended with fluid.

With respect to the *prognosis* in dropsy this our author very justly remarks depends on its causes and conditions. The anasarca occurring in chlorotic young women is the least dangerous—of the rest, febrile dropsies are more obedient to treatment, and oftener admit of complete recovery, than the passive or chronic. As far as the mere water is concerned in the chronic forms of the disease, cardiac dropsies are more readily dispersed for a time, but are more likely to return than dropsies complicated with renal disease. With respect to the principles of treatment laid down, they are at the same time very simple and very judicious—the first object is to get rid of the preternatural accumulation of watery fluid; the second, to prevent its collecting again: in other words, to remedy the diseased condition which gave rise to the dropsy. *Venæsection* will often reduce the dropsical swelling, more especially in active or febrile anasarca—its use seems to depend on its diminishing the congested state of the venous system—it tends to abate the undue action of the heart; and, by emptying the blood-vessels, it facilitates the re-absorption of the effused fluid and its ultimate expulsion from the system. Blood-letting, however, is not always applicable in cases of dropsical effusion. It is well known to have the effect of impoverishing the blood by robbing it of its fibrine; it also weakens the heart's powers, and thus has a tendency to produce muscular debility of this organ, which we have already seen to be one of the conditions on which the occurrence of dropsical effusion depends. When it is deemed inexpedient to draw blood, our effort should be to empty the vessels indirectly so as to remove from them their more watery parts only; that is, to promote the discharge of watery fluid from one or more of the secreting surfaces of the body; this is done, by acting on the kidneys, on the mucous surface of the alimentary canal, and also on the surface of the skin. Thus, the circumstances which are to determine our choice, require considerable reflection.

Having now followed our author through the first part of his work, in which he lays down the *General Principles of Pathology*, we shall proceed to the analysis of what he has to say on the nature and treatment of particular diseases. We shall commence with the diseases of the *Thorax*.

*Catarrh.*—We shall commence with this, the commonest of all thoracic complaints—it implies inflammation of the mucous membrane of the air-passages, and receives various names, according to the district of that membrane which it affects; gravedo in the frontal sinuses; coryza in the Schneiderian membrane of the nose; bronchitis in the trachea and lungs. The treatment of the trivial form of this disease is very simple—abstinence from animal food and stimulating liquor—remaining in an equable temperature—gentle aperients, and diaphoretics, include all that is necessary. Dr. Watson mentions the case of a fellow-student of his, who was very liable to catarrhal attacks, which he bore very impatiently. On one occasion, however, almost by accident, he took twenty drops of laudanum, just as one of his colds was beginning to torment him; and he found that the initiatory symptoms ceased. Since this time he has constantly had recourse to the opiate under similar circumstances, and finds himself quite well and comfortable in the course of half an hour after taking it. This, however, will not suit every person—plethoric persons should not adopt it. Dr. C. J. B. Williams has proposed what is called the *dry* plan of cure; it consists merely in abstinence from every kind of drink. No liquid, or next to none, is to be swallowed until the disorder is gone. The principle is that of cutting off the supply of watery materials to the blood. The wants of the system exhaust, from the circulating fluid, all that can be spared for the natural evacuations; and there is nothing left to feed the unnatural secretion from the inflamed mucous membrane. Its capillary vessels cease to be congested; the morbid flux is diverted, and the inflammation starved away. Dr. Williams assures us that a cure may be achieved in this way in forty-eight hours. This plan also has the advantage of not confining the individual to the house. The habitual use of the shower-bath is recommended by our author as an excellent *preventive* for those who are liable to colds. Its operation as a prophylactic seems to be this: it inures the surface to a lower temperature than it is likely to be subjected to in any other part of the day. Where a shower-bath cannot be conveniently had, cold sponging will be found very salutary. Inflammation of the membrane lining the air-passages may be, and often is, a very dangerous disorder; *i. e.* it may be both intense and extensive; it may descend in the vesicular texture, and occupy the whole surface of the membrane on one side of the chest; or it may involve the whole lining membrane of both lungs, and then there exists considerable danger. The treatment proper for acute and serious forms of bronchitis is thus stated by our author: if there be much fever, a hard pulse, and great oppression of breathing, and the patient be young, strong and robust, we must bleed him from the arm—this will at all events *relieve* the symptoms; but the bleeding must not be carried to syncope, nor repeated again and again, as a great part of the danger to be apprehended in the advanced period of the disease is, that the patient may not have muscular power enough to disembarrass his air-passages of the phlegm that overloads; to draw a strong breath and to achieve a vigorous cough. Sixteen ounces will be a moderate bleeding at first for an adult; the pulse, however, will afford a better guide for the abstracting of blood, than the local symptoms. *Topical* blood-letting over the surface of the chest or between the scapulæ is also to be recommended. After the bowels have been well cleared out by calomel and jalap, tartar emetic will be found a valuable medicine in acute cases of bronchitis. The depression which this substance produces is great, but it is temporary, and it is effected without loss of blood. Should symptoms of sinking and debility show themselves, stimulating expectorants must be administered. Carbonate of ammonia is thought to be a specific in such cases; five or six grains of it, given in solution every four or six hours, are often followed by free expectoration and marked improvement. Patients labouring under bronchitis are often harassed by severe cough and want of sleep. The employment of opium in such cases requires great caution indeed. Opium is, as our author well observes, a very ticklish remedy in such cases. He says he has known cases of patients, labouring under extensive bronchitis, who have been put so soundly to sleep by a dose of opium on going to bed that they have never waked again. He lays it down, as a golden rule in such cases, not to give opium—at least in a full dose, so as to force sleep—if you see any venous blood mingling in the general circulation,—if the complexion be dusky, and the lips in any degree blue. The circulation of half-arterialized blood through the brain, is in itself a powerful cause of coma; and, if you add the influence of an opiate, the coma may be made fatal. While the cheeks and lips remain florid, and when the first violence of the disease has abated, an opiate will do capital service. Counter-irritation, by a large blister laid across the chest, often affords sensible relief to the cough and oppressed breathing.

The author remarks how little disposed bronchitic inflammation seems to be to extend itself from the mucous membrane to the neighbouring tissues; and the reason assigned for this by our author is, that this membrane is furnished with a distinct set of blood-vessels, the bronchial arteries, and veins; while the substance of the lungs is supplied by the pulmonary. This perhaps is a mistake on the part of our author; both the mucous membrane, and the substance of the lungs, receive their nutrition from the bronchial arteries.

*Pneumonia.*—Inflammation of the substance of the lungs we shall now consider. There are three well-marked states of the lung, corresponding to different degrees and periods of its inflammation. The first is that wherein the substance of the lung is gorged with blood, or bloody serum. It is of a dark red colour externally, and crepitates less under pressure than the sound lung. In this state the lung is more easily torn; more, in that respect, like the spleen; and accordingly the term *splenization* of the lung has been given to this stage of its inflammation, as hepatization has to that which succeeds it. If the inflammation continue, the lung undergoes a further alteration: it no longer crepitates under pressure and it sinks in water; its cut surface presents sometimes a uniform red colour; sometimes a slightly mottled appearance, produced by an intermixture of the black matter of the lung and of the interlobular cellular tissue, which is less red than the other parts; the spongy character of the organ is lost; it is evidently solid; and the cut surface very much resembles the cut surface of the liver. Hence the term *hepatization*.

In a degree still further advanced, the pulmonary tissue, dense, solid and impervious to air, as in the last stage, undergoes an alteration of colour; it presents a reddish yellow, or straw, or drab, or stone colour; or it is of a greyish hue, sometimes mottled with red, or with the black pulmonary matter. It is full, in fact, of puriform matter, which is sometimes so abundant, that it oozes out plentifully, when incisions are made into the lung. The grey pus shows itself on the cut surface in the form of minute drops. This third stage has been called by Laennec *grey hepatization*, or *purulent infiltration;* Andral calls it *grey softening*. The author notices it as a very remarkable circumstance, that inflammation of the lung, going on to suppuration, does not lead to the formation of a circumscribed abscess, as it does when it affects the cellular tissue, or the parenchymatous tissue, in other parts of the body. Abscess of the lung is in fact a very rare thing. Laennec only met five or six cases of it, and Andral but one. Dr. Watson attempts to account for this, and refers it to the influence of the atmospheric air. First, he says, there is an effusion of serum and blood, then of lymph and blood; but the air passing into the surrounding sounder tissue, and mingling for a time even with the inflamed portion itself, causes the suppurative process to supersede the adhesive, and so no wall of circumvallation is formed by the coagulable lymph, as is the case in cellular tissue which is not accessible to the air.

Inflammation of the bronchi constantly accompanies inflammation of the parenchyma. When a single lobe is inflamed, it has been observed that the redness of the mucous membrane existed in those bronchial tubes alone which were distributed to that lobe. There may be bronchitis without pneumonia; but pneumonia without a corresponding extent of bronchitis is perhaps never seen. The majority of the cases of pneumonia are attended also with inflammation of the investing membrane of the lung; there is some pleuritis.

With respect to the treatment of pneumonia recommended by our author, it is precisely that usually adopted by all judicious practitioners; viz. blood-letting, tartar emetic, mercury. Of these he considers blood-letting the chief. In the first place, it extinguishes the inflammation *as* inflammation—in the next place, it has the effect of relieving the particular *function* of the lungs. The more blood is sent to them in excess, the more dyspnœa must there be, the more venous blood passing into the arteries, as well as the more risk of the effusion of lymph, and the obliteration of the cellular texture of the organ. When we bleed, says our author, in pneumonia, we kill two birds (as the phrase is) with one stone. We do that for the lung, which we do for an inflamed eye, when we darken the room, or for an inflamed joint, when we keep it at rest, *i. e.* we do all that we can to spare the exercise of the organ. The abstraction of blood will be effectual, *cæteris paribus*, in proportion as it is *early;* during the first stage—the stage of engorgement—and before the spongy texture of the lung has been obliterated. The patient should be bled in an upright position, by a large orifice and in a full stream; and the bleeding should be continued until some sensible impression is made upon the system: until the *pulse* becomes *softer;* or, if it were contracted, until it becomes *fuller;* until the *sensation* of constriction is abated, and the *dyspnœa* relieved; or until *syncope* appears to be at hand. The patient should be seen always within four or five hours after the first bleeding, that a timely *repetition* of it may take place, if the relief has not been complete, or *permanent*.

Our author likewise recommends the local abstraction of blood from the surface of the chest by cupping-glasses, or by leeches, especially if there be pain.

A time, however, arrives, when bleeding is no longer useful, when even it may exercise an injurious influence on the entire system, by reducing the patient's strength, and disabling him from bringing up and ridding his lungs of the tenacious mucus exhaled by the bronchial membrane. Some medicine is, therefore, wanted to assist the lancet, or to employ alone, when the lancet can do no more; there are two such, viz. *tartarised antimony* and *mercury*—the tartar-emetic being best adapted to the stage of *engorgement*, and the mercurial plan to the second stage—to that of *hepatisation*. When the second stage—that of solidification, has arrived, mercury is more worthy of confidence, than tartar-emetic. The object of giving it is to make the gums tender; and this should be done as speedily as possible. By these means the effusion of lymph, tending to spoil the texture of the lung, is arrested; and the lymph already effused begins to be again absorbed. We find that, from the great length we have gone in our analysis of this work, we must close our notice of it here for the present; not however without expressing our unqualified approbation of the manner in which Dr. Watson has performed his task. It cannot of course be expected that we should give any thing like a complete analysis of a work which embraces the entire province of practical medicine. The notices of the work which we have given are amply sufficient to enable our readers to form an accurate estimate of its general character, and to convey to them a just idea of the author's matter and manner. But it is as a book of elementary instruction, that we admire Dr. Watson's work. He is precisely the sort of person to write such a book; whilst he willingly acknowledges the benefits to be derived from the study of pathological anatomy to medical science, he carefully impresses on the mind of the student, that there are very many diseases in which morbid anatomy throws but little or no light on the nature of disease, especially in affections of the fluids and the neuroses—he insists on the great advantages to be obtained from the careful study of symptoms in those instances where it is impossible to connect those symptoms with diseased alterations of structure. In a word, Dr. Watson justly appreciates, and distinctly points out to the student, the great difference between the mere morbid anatomist, and the practical physician.

---

We now resume our analysis of Dr. Graves' truly interesting and valuable work on Clinical Medicine. We take it up at Lecture XXXII., in which he treats of *Sleeplessness*, as a result of disease. This state is sometimes observed to accompany certain morbid conditions of the system brought on by active disease, or by grief, care, and various other forms of mental disturbance, and frequently resists the most powerful and decided narcotics.

The first case noticed by Dr. Graves, in which this symptom was observed to occur, was a man who was just beginning to recover from jaundice—in jaundice everything denoting an unusual state of the nervous system, whether it be too much or too little sleep, is deserving of attention. In the case alluded to, the jaundice was the result of an attack of hepatitis, which had been treated with leeches, blisters, and mercury, after which, in the course of a few days, the stools became copiously tinged with bile, and his health seemed to improve. At this stage, the dejections being bilious, but the jaundice still remaining, the patient began to exhibit symptoms of restlessness and nervous irritability, and finally became perfectly sleepless. As a preliminary step Dr. Graves determined on evacuating the bowels, and for this purpose prescribed a purgative draught, consisting of five ounces of infusion of senna, half an ounce of sulphate of magnesia, a drachm of tincture of senna, and a scruple of electuary of scammony. His object was to purge briskly, and to give a full narcotic. He remarks that, in all cases of jaundice depending on hepatic derangement, after bilious evacuations have been procured, an active aperient should be prescribed every second or third day, for the space of ten days or a fortnight, with the view of carrying off the remains of the disease, so as to prevent a relapse. Such cases will be much improved by the use of Cheltenham water, taken every day for three or four weeks, *after the re-appearance of a bilious tinge in the alvine discharges.*

He here makes a practical remark well deserving of attention with respect to the mode of prescribing purgative mixtures consisting of infusion of senna—he says the quantity of this medicine usually given is much too small; he advises from three to six ounces of it to be given, where the patient's condition will admit of free purging. In chronic cases, too, purgatives should be administered at bed-time, and not, as is usually done, in the morning. In the above case of jaundice, after the purgative had acted well, he prescribed eight minims of black-drop to be taken at a late hour in the evening. With respect to this point, he gives a useful hint as to the time of giving opium to procure sleep; viz. to select the period at which Nature usually brings on sleep, and which varies according to circumstances and the habits of the patient. In dealing with watchfulness in patients labouring under morbid states of the constitution, inquire when the tendency to sleep usually occurs, and administer your narcotic about an hour or two before its occurrence. In these cases, to arrest the sleeplessness completely, one must persevere in the same plan of treatment for some days, until the tendency to sleep at a fixed hour becomes decidedly established.

Another disease in which sleeplessness is a prominent symptom, is delirium tremens. In such cases the Doctor has derived signal benefit from the combination of tartar-emetic and opium. There is one form of nervous irritability frequently observed in persons who are in the habit of drinking freely, but without running into excess, and presenting a shadow of delirium tremens. This state is observed to occur in persons about the middle period of life, who consume a larger quantity of spirits than they are able to bear. Such persons get into a chronic state of disturbed health, loss of appetite, impaired digestion, an irritable state of the nervous system, and watchfulness. To relieve this state Dr. G. recommends a mixture which he has found very beneficial, viz. tincture of columba, quassia, gentian, and bark—of each one ounce; to this is added a grain or even two, of morphia. The dose of this mixture is a tea-spoonful three or four times a day—the best time for taking it is about one hour before meals—the state of the bowels should likewise be attended to.

Another disease in which sleeplessness occurs as a very unmanageable symptom, is fever. The most successful mode of treating this symptom was found to be opiates in the form of injection. Baron Dupuytren was the first who made this important observation, and proved that narcotics applied to the mucous surface of the rectum exercise powerful influence on the nervous system, always equal, and very often superior, to the effect produced by taking them into the stomach.

The delirium and sleeplessness arising from the irritation of blisters is by no means uncommon—it chiefly occurs in the case of children, in whom the cutaneous surface is very irritable. Such cases should be treated with opium, given in small but frequently repeated doses, so as to insure its energetic, but safe action—the child should also be prevented from scratching the blistered surface.

The next kind of sleeplessness to which the Doctor directs attention is that which is frequently met with in persons of a nervous and irritable disposition, in hypochondriacs and hysterical females. In the latter subjects, more especially, antispasmodics and remedies possessing a stimulant effect, are those from which he has found most benefit. Musk, either alone, or combined with assafœtida, were found to be the most successful.

On the subject of applying cold lotions to the shaved scalp in affections of the head occurring in acute diseases, and attended with raving and loss of rest, the author makes some excellent practical remarks. Cold lotions, as they are usually applied, produce effects the very reverse of that intended: and that because they are applied at such long intervals, as to allow the scalp to re-act, and resume its heat. Dr. Graves advises, when we wish to apply cold with effect, that we should have it done by relays of folded linen, wet with any frigorific mixture, and repeatedly applied to the scalp, so as to leave no smoking, or, what is much better, get three or four bladders, put into each a quantity of pounded ice, and apply one over the crown of the head, one on each side, and lay one on the pillow for the back of the head to rest on.

In Lecture XXXIII. Dr. Graves treats of Inflammation and the motor powers, which cause and regulate the circulation, and more especially the capillary circulation. The perusal of this lecture will amply repay the attentive reader—indeed the subject has a bearing so immediate on one of the most important questions in pathology—the nature of inflammation—that it deserves the fullest attention. The chief question in dispute is the degree in which the capillary circulation is influenced by any other agency than the contractile power of the heart and arterial system;—some physiologists maintaining that this alone is sufficient to account for the capillary circulation; and others asserting that it is necessary to admit some supplementary force, which may either assist, retard, or regulate the flow of blood from the arteries into the veins. We must decline any attempt at the analysis of this admirable lecture, and pass on to the next, in which the author treats of certain forms of Gout. He premises some observations on constitutional inflammation in general. There is no proposition, he says, in pathology, better established than that there exist certain constitutional affections capable of generating and modifying local inflammatory action ; and that local inflammations, depending on a constitutional cause, are subject to very different laws from those which regulate the phenomena of common inflammation. There is one fact connected with local inflammation depending on a constitutional cause not sufficiently noticed, namely, that certain affections of this kind are sometimes remarkably fugitive and transient. Persons are accustomed to regard the process of inflammation, whether common or specific, as one which generally lasts for some days ; it occasionally happens, however, that a peculiar diathesis will give rise to local affections having the characters of inflammation which run their course and terminate in the space of a few hours. This observation, which should be borne in mind in the investigation of diseases connected with the general habit, will serve to explain some of the anomalies which strike us occasionally in the study of constitutional maladies. Scrofula is one of those constitutional maladies, which produces inflammations of various parts and tissues of the body, which last but a short time. Gout is another. Persons of a gouty habit are subject to sudden pains or twitches, which last only for a few minutes, or even seconds—these pains are seemingly the result of a momentary congestion. Thus, in various neuralgic affections, and in inflammatory diseases in which the nerves are considerably engaged, pain is suddenly produced by coughing, and this is occasioned by local congestion ; this is evident from the redness of the face, the hæmorrhage from the nose or from recent wounds, so often produced by a fit of coughing. From the fact that momentary congestion is capable of producing momentary pain, it may be inferred that gouty twitches are owing to some cause which determines an instantaneous congestion of the affected part.

Among the anomalous local affections connected with the gouty habit, Dr. Graves notices the morbid habit of grinding the teeth which some persons have. This grinding of the teeth continues for years as a daily habit, and produces very remarkable changes in the conformation of those organs, affecting sometimes one side of the jaw, sometimes both. Dr. Graves is satisfied that the irritable state of the dental nerves, which gives rise to this irresistible tendency to grind the teeth, depends chiefly on the existence of gout in the constitution.

Generally speaking, a regular attack of gout in the extremities is preceded by a longer or shorter period of constitutional disturbance and dyspepsia. We must not, however, in making the diagnosis between gout and rheumatism, consider this distinction as not liable to exceptions—the arthritic attacks have been known to come on suddenly, without the slightest precursory derangement of the health, or the operation of any assignable cause. Another exception to the general rule, as mentioned by the author, is deserving of notice. In general, a fit of the gout is preceded and accompanied by a scanty secretion of turbid high coloured urine. As the fit goes off, the urine increases in quantity, becomes clearer and paler, and loses its tendency to deposit the lithates and purpurates. The author has seen this order reversed. It is generally admitted that the gouty diathesis may excite its specific inflammation in most of the tissues of our organs. Our knowledge, however, regarding its effects in these various tissues is far from accurate. The changes it produces in the secretions of mucous membranes is a question which has not received sufficient attention. Though all admit the existence of gouty cough or bronchitis, the diagnosis and history of this affection are still very incomplete. The effects of gout on the lining membrane of the urethra and bladder are better known. Dr. Graves makes a remark here deserving attention concerning the bronchitis and pneumonia which accompany pulmonary consumption. He thinks that too much importance has been attached to the tubercles in this disease. What others call tubercular pneumonia, he would designate scrofulous pneumonia accompanied by tubercles. He is satisfied, in fact, that the essential characteristics of phthisis pulmonalis are derived from scrofula. This it is, which converts what would be common into consumptive pneumonia or bronchitis; this it is, which so often renders both incurable. Tubercles and tubercular infiltration are mere results of nutrition morbidly modified by scrofula; they are effects, not causes; they often exist without scrofulous inflammation, and the latter may exist without them.

Dr. Graves next directs our attention to what he calls "gouty ramollissement of the spinal cord." He states that, in certain cases, where gout attacks the nerves, giving rise to gouty congestion or inflammation, frequently recurring and acquiring increased strength and deeper root as it proceeds, the morbid affection may, after years or even months, run on until it reaches the spinal cord, involving a certain portion or portions of that organ, and producing loss of sensation and motion commensurate to the amount of spinal derangement. This is no uncommon occurrence; it is merely an instance of disease originating in the periphery of the nervous system, passing along the trunk of the affected nerve with a retrograde motion, and finally reaching the central parts, and then giving rise to various forms of paralysis. Dr. Graves has frequently, in his writings, proved that disease commencing in the nerves of some particular part or organ, may be gradually propagated to the spine, producing all the symptoms which are referrible to an original affection of the nervous centres. In various parts of his Clinical Lectures he has brought forward numerous facts in proof of the propagation of disease from the circumference to the centre of the nervous system. He has shewn, in fact, that enteritis, arising suddenly from indigestion and obstruction caused by an error in diet, was followed by paraplegia. He has treated cases of paraplegia occasioned by stricture, which were relieved by curing the stricture; he has also seen cases of acute and chronic affections of the uterus and kidneys which had entailed on the patients, as a remote consequence of the original disease, loss of the power of motion in the lower extremities, sometimes partial and curable, sometimes irremediable and complete. He has, in a word, seen cases which warrant him in concluding that gouty inflammation of the nerves and their neurilema, may, in process of time, extend to the spinal marrow and its investments, and give rise to derangements of the latter, terminating in ramollissement and structural degeneration. When paraplegia originates in disease of the spinal cord itself, retention of urine, or irritability of the bladder, often announce the approach of the disease long before the loss of power in the limbs becomes evident; whereas, in all those cases in which the paralysis creeps from the extremities along the nerves towards the spinal marrow, the bladder is affected only at a late period of the disease. It is obviously of great importance that practitioners should be aware of this termination, as a knowledge of the fact may lead them to the timely adoption of preventive measures. Dr. Graves having experienced the total inefficacy of colchicum, hydriodate of potash, strychnine, and all the usual remedies, recommends the early insertion of issues over the spine, with prompt and decided mercurialisation. It may be well to observe that gout may, without first attacking the nerves of the extremities, attack the spinal marrow and its investing membranes in the first instance, or in consequence of metastasis.

Dr. Graves now makes some valuable, because practical, remarks on the effects of Mercury in Scrofulous Affections of the Lung. He was first led to the adoption of this mode of treatment by the successful results of mercury in the hands of Dr. O'Beirne in treating scrofulous inflammation of the joints. This employment of mercury in the treatment of scrofulous diseases was directly in the teeth of the prevailing theories of the day, according to which mercury was not only inadmissible, but absolutely mischievous in persons of a scrofulous diathesis. Dr. Graves was led by analogy to apply the same principle of treatment to incipient scrofulous inflammation of the lung. He points out the cases to which such treatment is applicable; it is, he says, only in those cases wherein scrofulous inflammation commences in the lung before any general contamination of the system has taken place, that mercury ought to be tried, and it will be of no avail except where the commencement of the scrofulous inflammation of the lung has arisen suddenly, and in consequence of the operation of some obvious cause, as catching cold, or the occurrence of hæmoptysis. Dr. Graves thinks, that too much stress has been laid on the affection of the lung by writers on phthisis. In most cases, no doubt, the disease commences in the lung; but, in other cases, it passes through many changes, and affects various organs before it attacks the lung. Dr. Graves has a decided objection to the terms "tubercular inflammation," and considers the whole theory of inflammation being excited in the lung by the presence of tubercles, to be founded on erroneous views. He considers, also, that tubercles do not act in all cases as foreign bodies. He thinks that, in the treatment of scrofulous bronchitis and scrofulous pneumonia, mercury is a most valuable remedy—where the attack is recent, and has occurred under circumstances which preclude any suspicion of previous tubercular disease.

Dr. Graves makes some very useful remarks on a form of chronic laryngeal inflammation, which has been described under the name of phthisis laryngea. Of this disease he notices two varieties. In one case the hoarseness and sore throat follow the development of tubercles in the lung; in the other they precede it. Consumptive persons very frequently get, shortly after the occurrence of scrofulous inflammation of the lungs, sore throat, hoarseness, and laryngeal cough. But this is different from the hoarseness and cough which precede phthisis. In the former, the laryngeal symptoms are secondary, and form only a part of the general disease; in the latter, they constitute the first link in the chain of morbid action. The former takes place only in a constitution decidedly scrofulous; the latter occur most commonly in constitutions enfeebled by various debilitating causes. Now, the order of succession may be very easily inverted, and on such a constitution the accidental circumstance of a cold falling on the larynx, may determine the appearance of disease in that part long before the lungs become engaged. Hence, in a case of chronic laryngitis, where the disease has lasted for any length of time, and where the patient's system has been impaired by any debilitating cause, or where scrofula is suspected, the prognosis must be guarded. Should there be no scrofulous deposition in the lung, the case is not to be given up at once. First, remove the inflammation of the throat, if possible—then endeavour to improve the state of the constitution. If there be much tenderness of the larynx on pressure, commence with the local detraction of blood. A few leeches are to be applied to the throat every second or third night: this to be continued for a week or fortnight. If there be no tenderness, leeches are not required. Then direct attention to those means which act immediately on the diseased mucous surface: one of the best applications for this is, a solution of nitrate of silver, or of sulphate of copper (10 grains to the ounce). The object of this is to change the action of the mucous surface. Iodine inhalations will also be found useful. Counter-irritation also, as by croton-oil frictions, will be found useful in this case. The use of decoction of sarsaparilla, with nitric acid, will also be found useful in these cases. The patient also must refrain as much as possible from speaking.

*Hoarseness.*—Dr. Graves makes some very judicious remarks on a form of hoarseness frequently observed in growing boys or girls, which assumes a very chronic character, and often resists every form of treatment. A boy gets cold, followed by sore-throat, and feverish symptoms, which may last for a few days, and then disappear under the use of aperient medicines, or perhaps without any interference. The feverishness and soreness of throat subside, but the hoarseness remains. Everything else may go on perfectly well, with the exception of this impairment of the voice.

On examination there is found no appearance of inflammation in the mucous membrane, no tenderness in the region of the larynx. This form of disease depends on a relaxed state of the chordæ vocales, and, perhaps, the muscles of the larynx. In such cases Dr. Graves recommends the use of strong stimulant gargles—tincture of capsicum with decoction of bark—frictions over the region of the larynx and external fauces, with croton oil—strict silence should be enjoined. Should these means fail, the inhalation of vapour arising from tincture of iodine and tincture of conium, added to hot water in a proper apparatus, may be employed; but in all obstinate cases the sheet-anchor is mercury exhibited internally, and by means of inhaling the fumes of hydrargyrum cum cretâ. The mouth should be slightly touched. Before we employ mercury, however, for the cure of hoarseness, we must ascertain whether it arises from a phthisical tendency.

We now find ourselves compelled to terminate our notice of Dr. Graves's work. Before doing so, however, we deem it but justice to state, that we consider it as one of the most valuable contributions that has been made for many years to practical medicine. The excellent, practical points to be found in every page of the book, fully entitle Dr. Graves to the high character he has long sustained, that of a sound, well educated physician, who, to talents evidently of the very first order, has superadded all the advantages to be derived from cultivated experience, and an intimate acquaintance with the works of the ablest medical writers, both foreign and domestic. To the untiring energy, with which Dr. Graves has applied these great advantages in establishing a school of Clinical Instruction, the Dublin School of Medicine is mainly indebted for the high character it now enjoys all over Europe and America. Of the characteristic originality of Dr. Graves's mind we have had many proofs in the numerous discoveries and improvements made by him in the field of pathology; nor is that the least wherein he establishes, by numerous facts, that *disease commencing in the nerves of any particular part or organ, may be gradually propagated to the spine, and so produce all the symptoms referrible to an affection originating in the nervous centre.* We cannot, for the life of us, help thinking that something more than the mere germ of the reflex-function-principle is contained in this proposition, laid down and proved by Dr. Graves many years since—nor would this be the first instance wherein physiology was indebted for its advancement to the helping hand of pathology.

